# The Role of Interleukin-1 and Interleukin-18 in Pro-Inflammatory and Anti-Viral Responses to Rhinovirus in Primary Bronchial Epithelial Cells

**DOI:** 10.1371/journal.pone.0063365

**Published:** 2013-05-28

**Authors:** Siân C. Piper, John Ferguson, Linda Kay, Lisa C. Parker, Ian Sabroe, Matthew A. Sleeman, Emmanuel Briend, Donna K. Finch

**Affiliations:** 1 Respiratory, Inflammation and Autoimmunity, MedImmune Ltd, Cambridge, United Kingdom; 2 Department of Infection and Immunity, Faculty of Medicine, University of Sheffield, Sheffield, United Kingdom; University of Georgia, United States of America

## Abstract

Human Rhinovirus (HRV) is associated with acute exacerbations of chronic respiratory disease. In healthy individuals, innate viral recognition pathways trigger release of molecules with direct anti-viral activities and pro-inflammatory mediators which recruit immune cells to support viral clearance. Interleukin-1alpha (IL-1α), interleukin-1beta (IL-1β) and interleukin-18 (IL-18) have critical roles in the establishment of neutrophilic inflammation, which is commonly seen in airways viral infection and thought to be detrimental in respiratory disease. We therefore investigated the roles of these molecules in HRV infection of primary human epithelial cells. We found that all three cytokines were released from infected epithelia. Release of these cytokines was not dependent on cell death, and only IL-1β and IL-18 release was dependent on caspase-1 catalytic activity. Blockade of IL-1 but not IL-18 signaling inhibited up-regulation of pro-inflammatory mediators and neutrophil chemoattractants but had no effect on virus induced production of interferons and interferon-inducible genes, measured at both mRNA and protein level. Similar level of virus mRNA was detected with and without IL-1RI blockade. Hence IL-1 signaling, potentially involving both IL-1β and IL-1α, downstream of viral recognition plays a key role in induction of pro-inflammatory signals and potentially in recruitment and activation of immune cells in response to viral infection instigated by the epithelial cells, whilst not participating in direct anti-viral responses.

## Introduction

Significant unmet medical need remains for the reduction of frequent or severe acute exacerbations (AE) in patients with chronic respiratory disease, such as chronic obstructive pulmonary disease (COPD). Patients with moderate to severe COPD and a history of exacerbations continue to have frequent and severe exacerbations despite treatment, have a worse quality of life and an increased risk of mortality [1], [2], [3], [4]. Human rhinovirus (HRV) is a very commonly detected virus at exacerbation [5], [6], and has been associated with higher AECOPD symptom scores [6]. Host responses to rhinovirus appear aberrant in COPD patients [7] and therefore further investigations into the mechanisms involved in viral recognition and pro-inflammatory responses is required to inform similar studies in AECOPD.

HRV is a non-enveloped single stranded RNA virus of the *Picornaviridae* family which predominantly and initially infects cells of the airways epithelium [8]. HRV serotypes are principally major or minor group viruses which bind to intracellular adhesion molecule-1 (ICAM-1) or low-density lipoprotein (LDL) receptor respectively, and there is also a group of HRV-C viruses for which the mode of infection is unknown [8]. Following HRV infection, epithelial cells release inflammatory mediators which activate lung-resident macrophages and together recruit immune cells required for optimal viral clearance. These mediators include those that amplify local inflammation (such as IL-1), mediate specific patterns of leukocyte recruitment and activation (such as IL-8, IP-10, IL-6) as well as those that initiate anti-viral defence (such as interferons (IFN) IFNβ, IFNλ) [9].

Although much is known about the role of pattern recognition receptors in host anti-viral defence, host recognition of HRV infection is not yet fully understood. Rhinoviral detection involves the pattern recognition receptors MDA-5, RIG I, TLR3, and interferon-inducible elements [10] and also TLR7/8 [11], working in a co-ordinated fashion. Unlike most cytokines, IL-1β and IL-18 are translated without a leader sequence, resulting in their accumulation within the cytosol [12]. Activation of multi-protein complexes known as inflammasomes, results in initiation of caspase-1 mediated cleavage of pro- IL-1β and pro-IL-18 into their mature forms, allowing their secretion [13]. Antiviral immunity involving the NLRP3, AIM-2 or RLRs, can result in the assembly of inflammasomes, thus linking viral sensing with release of IL-1β and IL-18 [14], [15] although this has not been specifically elaborated for HRV. In addition to viral nucleic acid recognition, other pathogen-associated molecular patterns and virally-induced signaling events can also contribute to the inflammatory response. For example, activation of spleen tyrosine kinase (Syk) downstream of ICAM binding of major group viruses has been implicated with cytokine release after HRV infection [16]. Increases in pro-inflammatory mediators are seen with replication deficient virus, indicating rapid viral recognition immediately following infection [17], [18]. Knockdown of Syk resulted in a partial reduction of IL-8 in response to HRV infection, suggesting that multiple mechanisms of IL-8 induction combine [16].

It is possible direct cell death following viral infection may contribute to the inflammatory response. Both IL-1β and IL-18 can be processed to their active forms by several soluble proteolytic enzymes if the pro-cytokines are released from the cytosol of cells [19], [20], for example upon uncontrolled forms of cell death or lysis. Although IL-1α can be cleaved, such cleavage is not mediated by caspase-1 and IL-1α does not need to be processed to be active, but passive release of IL-1α from dead cells can induce inflammatory processes in response to necrosis [21]. IL-1α and IL-1β may recruit different myeloid cells at different stages of sterile inflammation [22] suggesting that release of and response to these related molecules can be substantially co-ordinated.

Given that IL-1α, IL-β and IL-18 are highly related cytokines, we sought to understand their role in normal epithelial responses to HRV with greater granularity. Although well understood that IL-1 is an important cytokine in the establishment of inflammation, it is not known specifically whether host recognition of HRV results in active or passive release of IL-1, whether caspase-1 activation is required and what the relative role of IL-1 and IL-18 are in amplifying specific epithelial cell responses. The production of these cytokines was studied, with particular regard to caspase-1 processing and cell death, in response to HRV infection of primary normal human bronchial epithelial cells. Protein and gene-modulation of known components of anti-viral immunity were investigated in the presence of inhibitors of signaling of these cytokines with HRV infection.

Although release of active IL-1β and IL-18 was dependent on viral entry and caspase-1 cleavage, IL-1α release was dependent on viral entry, but not dependent on cell death or caspase-1 catalytic activity. A role for IL-1α in direct viral responses has on the whole been overlooked until now. Whilst inhibition of IL-18 in this assay system had no effect, blockade of IL-1RI had a clear impact on classic pro-inflammatory responses of epithelial cells, but did not impact interferon and interferon-inducible pathways, and did not alter viral load.

## Materials and Methods

### Inhibitors

All inhibitors used were commercially available: anti-ICAM-1 mAb (clone 14C11 R&D Systems, Minneapolis, MN), YVAD (VWR, Lutterworth, UK), BAX inhibitory peptideV5 (Sigma, St Louis, MO), IL-18BPa-Fc chimera (R&D Systems, Abingdon, UK), anakinra (commercially available Kineret®, Amgen/Biovitrium).

### HRV production

Virus was propagated by infection of Hela-H1 cells (American Type Culture Collection (ATCC), LGC Standards, Teddington, UK) with HRV14 (ATCC) for 48 h at 33°C in EMEM containing 2 mM L-Glutamine and 1% non essential amino acids (NEAA). Virus-containing supernatant was collected, centrifuged, and virus precipitated with 7% PEG6000 and 0.5 M NaCl at 4°C overnight. Following precipitation virus was centrifuged and resuspended by overnight incubation in PBS. This was then centrifuged, filtered (0.22 μm Steriflip filter (Millipore, Watford, UK)) and concentrated (100,000 NMCO centrifugal filter device (AMICON, Watford, UK)). During the concentration step buffer was exchanged to fresh PBS in order to remove any contaminating cytokines.

**Figure 1:**
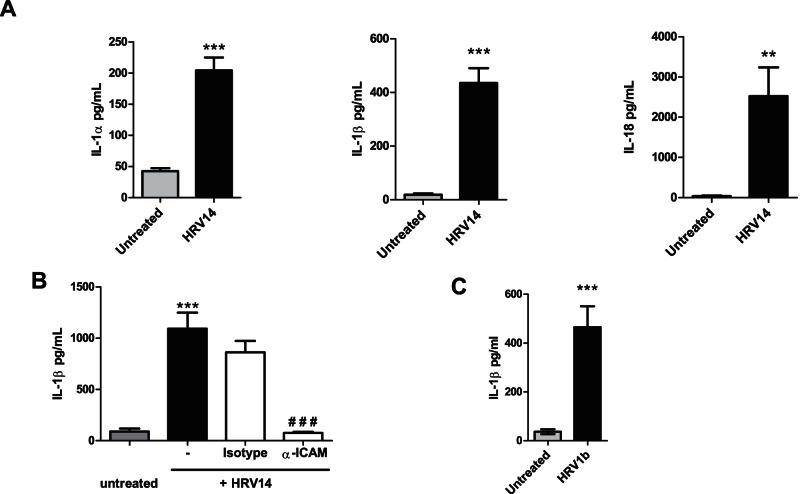
HRV induces the release of IL-1α, IL-1β and IL-18 by NHBE cells. NHBE cells were infected with or without HRV14 **(A and B)** or HRV1b **(C)**. After 48 h the levels of IL-1α **(A)**, IL-1β **(A, B and C)** and IL-18 **(A)** released into the media were measured by ELISA. In **(B),** some samples were treated with an anti ICAM-1 antibody or an isotype control (both 1.33 μM) for the duration of the infection. Data is expressed as mean ± SEM. An * indicates a significant up-regulation in protein release, a **#** indicates there was a significant inhibition in protein release (***/^# # #^ *p*<0.001, **/^# #^ *p* = 0.001–0.01, */^#^ *p = *<0.05). *N* = 8 **(A)**, 7 **(B)** or 4 **(C)**. Untreated indicates cells were treated with medium alone.

Median tissue culture infective dose (TCID_50_) of purified HRV14 was determined by incubating Hela-Ohio cells (European Collection of Cell Culture (ECACC), Porton Down, UK) (3×10^4^ cells seeded in a 96 well plate 24 h before infection) with 10 fold serial dilutions of virus in MEM containing 100 units/ml of each of penicillin and streptomycin, 1% NEAA, and 10% FCS (Invitrogen, Paisley, UK). Cells were incubated with the virus for 6 days before they were fixed in 3.7% formaldehyde, and stained for 10 minutes in 0.1% crystal violet. The mean absorbance of 100 scans at 600 nm was calculated per well. All wells with average absorbance less than 80% of non-infected controls were considered to be infected. After scoring the number of infected wells (of a maximum of 6 wells) at each titration of virus the Spearman Karber method was used to calculate the TCID_50_ [23]. This was then used to calculate the multiplicity of infection (MOI, the number of infectious units/cell). Sample data and calculation is shown in Table S1. Normal human bronchial epithelial (NHBE) cell cytokine release measured at 48 h after 3 h viral incubation was significantly different from uninfected cells at a low and higher MOI (Figure S2) and was ICAM-1 dependent (Figure 1B). Based on these data an MOI of 0.007 was used for all experiments.

### NHBE cell culture and infection

NHBE cells (Lonza, Zurich, SW) from 3 different donors were cultured in bronchial epithelial basal medium (BEBM) containing bronchial epithelial growth medium supplements (BEGM) (Lonza). NHBE cells (passage 2) were seeded at a density of 1.2×10^5^/ml and incubated overnight. The next day, incubation with virus and appropriate inhibitors progressed for 3 h at 33°C before the virus-containing medium was replaced with BEGM medium containing inhibitors alone. Cells were incubated for 48 h, unless indicated otherwise, before the media was collected for mediator analysis by ELISA. If required for RNA extraction, cells were washed in PBS, lysed in Qiazol (Qiagen, Crawley, UK) and lysates stored at −80°C until processed.

### Cytokine detection

All ELISA kits or detection/capture antibodies were purchased from R&D Systems. Pro-IL-1β was measured using a Quantikine ELISA kit according to manufacturer’s instructions. Interleukin-18 levels were determined using clones 125-25 and biotinylated 159-12B as capture and detection antibodies respectively. Duoset^TM^ ELISA kits for IL-1α, IL-1β, IL-8, IL-6 and IP-10 were used according to the manufacturer’s instructions except for a final detection step modification to a dissociation-enhanced time-resolved fluorescence method (DELFIA; all reagents Perkin Elmer, Boston, MA). This utilised Europium-labeled streptavidin diluted 1/1000 in DELFIA assay buffer (in replacement for streptavidin-HRP), and thorough washing and detection of time-resolved fluorescence (e.g. by use of proprietary Enhancement solution) as recommended by the manufacturer.

To detect pro-IL-18, media taken from infected NHBE cells or lysates from permeabilised NHBE cells (PBS containing 0.1% saponin and 1× complete protease inhibitors (Roche Applied Science, Burgess Hill, UK) were incubated with 0.2 units of recombinant Caspase-1 (Sigma Aldrich) in 1x reaction buffer (50 mM NaCl, 50 mM Hepes (pH 7.3), 10 mM EDTA, 5% glycerol, 0.1% CHAPS, 10 mM DTT) at 37°C for 1h. Samples were then diluted 1/10 into PBS containing 1% BSA before IL-18 levels were measured as indicated above.

### Cell death

Cell death was measured using a Cytotox-Glo kit (Promega, UK), which utilises loss of membrane integrity to indicate cell death, according to manufacturer’s instructions. Percentage cell death was quantified by permeabilising parallel wells of NHBE cells to define 100% cell death, as recommended in the kit.

### RNA purification and gene analysis

RNA was purified from Qiazol lysed NHBE cells using a mRNeasy mini kit (Qiagen) and cDNA was transcribed using a high capacity RNA-to-cDNA kit (Applied Biosystems, USA), both according to manufacturer’s instructions. IL-8 gene expression was analysed using IL-8 (Hs99999034_m1) and 18s (Hs99999901_s1) Taqman probes (Applied Biosystems) and amplified using Taqman Universal PCR MasterMix. A custom-made microfluidic genecard (Applied Biosystems) was used to analyse the effect of HRV14 infection on the host response, and contained a panel of 45 genes of interest. Results were obtained for both methods according to manufacturer’s instructions and using an ABI7900HT real time PCR system (Applied Biosystems). Expression was analysed using the 2^−ΔΔCt^ method (RQ Manager, Applied Biosystems) using 18s as the internal control. For most genes the data was expressed in relation to media control (uninfected cells) samples at the indicated time point. For several genes (*IFNB1, IL28A* and *IL-29*) there was no reproducible expression in the uninfected cells; to analyse the effect of IL-1/IL-18 blockade, these genes used sample treated with HRV14 alone as the reference condition. Viral copy number was assessed as previously described [24].

### Statistics

Where appropriate the data was analysed using Student's T-test or a One-way ANOVA with Bonferroni’s post test.

### Supporting information

Materials and methods for data described in Supporting Information is given in Materials and Methods S1.

## Results

### Human Rhinovirus infection of Normal Bronchial Epithelial cells induces the release of IL-1 and IL-18

The release of IL-1α, IL-1β and IL-18 was evaluated on rhinoviral infection of NHBE cells. A low MOI (0.007) of virus was used for two reasons; firstly to mimic the *in vivo* infection of human epithelium, occurring after inhalation of a very low number of virus particles [25]; secondly, to limit cytopathic effects that may occur following infection *in vitro* (see also **Figure S2**). After viral infection, the amount of IL1α, IL-1β and IL-18 present in the cell medium was measured by ELISA. A significant increase in the amount of all three cytokines could be detected in the supernatant of HRV-infected cells compared to control uninfected cells (**Figure 1A**).

HRV14 binds ICAM-1 on the cell surface and uses this adhesion molecule as a receptor to infect cells. Although we used a purified stock of virus, thus reducing the chance of cytokine contamination from cells used to produce the virus, we used a neutralising anti-ICAM-1 antibody to demonstrate dependence of the responses on viral binding. The release of IL-1β (**Figure 1B**) and IL-8 (data not shown), were inhibited in the presence of the anti-ICAM-1 antibody showing that infection of NHBE cells by HRV14 was required to trigger a measurable cytokine release.

The cytokine response is not specific to HRV14 as it could also be observed with a minor group strain, HRV1b (**Figure 1C**).

### IL-1β, IL-18 and their respective precursors are released by HRV-infected cells

**Figure 2:**
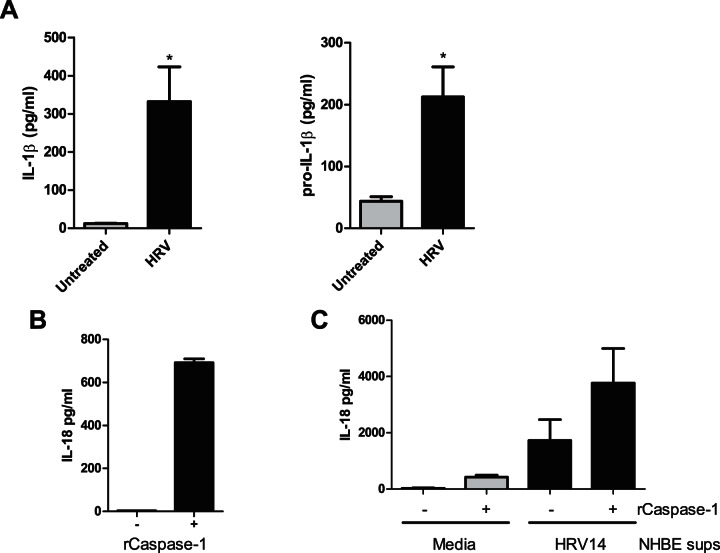
HRV14 induces the release of the pro- and mature forms of IL-1β and IL-18. **(A)** NHBE cells were infected with or without HRV14 and the levels of IL-1β and pro-IL-1β released into the media after 48 hours was measured by ELISA. Untreated indicates cells were incubated with medium alone. Data is expressed as mean ± SEM, an * indicates a significant up-regulation in protein release (IL-1β, *p* = 0.0168. pro-IL-1β, *p* = 0.0151.), *N* = 6. The levels of IL-18 in NHBE lysates (**B**) or media taken from NHBE cells (**C**) was measured by ELISA before and after treatment with rCaspase-1 in order to detect the presence of pro-IL-18. The data in (**B**) is expressed as mean ± SEM and is a representative graph from 3 independent experiments, the data in (**C**) is mean ± SEM (*N* = 3).

To determine what proportion of IL-1β and IL-18 was mature processed cytokine, and/or whether pro-forms could be detected in the supernatant of HRV-infected NHBE cells, we first assessed 2 different ELISA described as specific for either pro-IL-1β or IL-1β respectively. As expected, the pro-IL-1β kit detected pro-IL-1β and not mature IL-1β. However the IL-1β ELISA, whilst accurately measuring recombinant IL-1β, was also able to detect recombinant pro-IL-1β with 10% efficiency (data not shown). As shown (**Figure 2A**), both pro- and mature forms of IL-1β could be detected in the supernatant of HRV-infected cells obtained from 2 independent donors. A similar strategy could not be used to detect pro-IL-18 as there is no specific ELISA available. Instead recombinant caspase-1 was used to cleave the pro-peptide from pro-IL-18 and an ELISA specific to the mature form of IL-18 was then used to detect this newly processed mature IL-18. To validate this approach, uninfected NHBE cell lysates were first incubated with or without recombinant caspase-1 before the levels of IL-18 were determined, using the IL-18 ELISA. Mature IL-18 could be detected in the lysate treated with recombinant caspase-1 whilst there was no detectable signal in absence of enzyme. These data are in keeping with previous results showing preformed pro-IL-18 in cells [26], and this result validates this approach as a way to reveal the presence of pro-IL-18 in biological samples (**Figure 2B**). We then treated supernatants from infected and uninfected NHBE cells with recombinant caspase-1. As expected from experiments reported in figure 1A, in absence of caspase-1, mature IL-18 could be detected in the supernatants of HRV-infected cells (**Figure 2C**), but not uninfected cells. The signal from infected cells was further increased following caspase-1 treatment indicating that the precursor form of IL-18 is also being released.

### Virus-induced IL-1β and IL-18 release is an active process depending on caspase-1 activation

**Figure 3:**
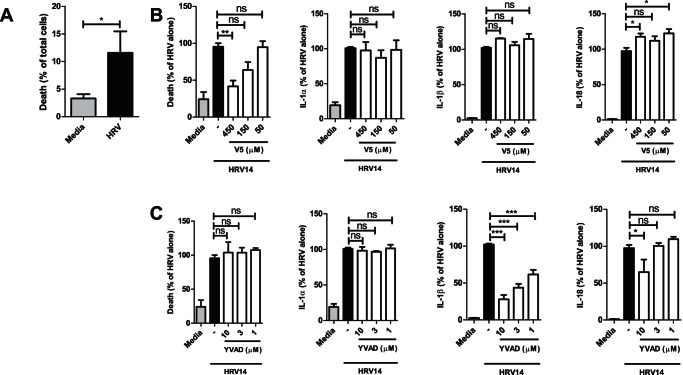
IL-1β and IL-18 are released by an active caspase-1 dependent event. NHBE cells were infected with or without HRV14 (**A–C)** in the presence of the V5 Bax inhibitory peptide **(B)** or the caspase-1 inhibitor YVAD **(C)** at the indicated concentrations. A – indicates treatment with the vehicle control. After 48 hours the level of cell death or cytokine release was measured. In B and C individual experiments were normalised by expressing each replicate as a percentage of the median signal detected in the HRV treated sample. Data shown is the Mean percentage ± SEM (*N* = 3).

Even at the relatively low MOI used in our experiments we observed an increase in cell death after HRV14 infection (from 3% to 12%) (**Figure 3A**). Therefore, we assessed the contribution of cell death and/or caspase-1 activation on the release of IL-1β and IL-18. Treatment with the BAX inhibitory peptide V5 during the infection inhibited the induction of cell death by HRV14 but had no effect on the release of IL-1β and IL-18 (**Figure 3B**). In contrast to this, treatment with the caspase-1 inhibitor YVAD had no effect on cell death but reduced the amount of extracellular mature IL-1β and IL-18 detected (**Figure 3C**). IL-1α release was not affected by the Bax V5 inhibitory peptide or the YVAD inhibitor **(** **Figure** **3B–C** **)**. Infection of NHBE cells by HRV is thus likely associated with recognition of viral products, oligomerisation of the inflammasome resulting in caspase-1 activation and release of mature IL-1β and IL-18, but also an alternative active process resulting in IL-1α release.

### Autocrine IL-1 signaling contributes significantly to IL-8 and IL-6 release, but does not alter viral load

**Figure 4:**
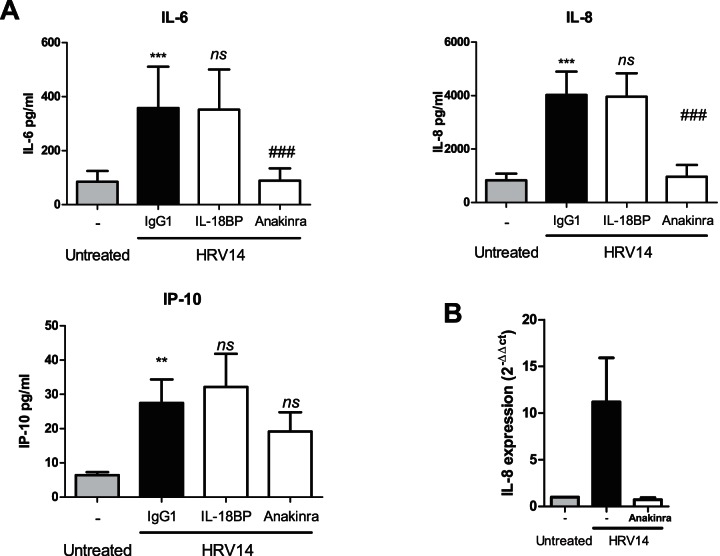
IL-1 is required for the HRV dependent release of IL-6 and IL-8 but not IP-10 in NHBE cells. **A)** NHBE cells were infected with HRV14 in the presence of a control IgG1 antibody (33 nM), IL-18BP-Fc (33 nM) or anakinra (10 nM). A – indicates no inhibitor treatment. The levels of IL-6, IL-8, and IP-10 released into the media after 48 hours was assessed by ELISA (*N* = 5). Data is expressed as Mean ± SEM, **B)** Levels of IL-8 mRNA were determined in NHBE cells that were infected with HRV14 for 24 hours in the presence or absence of anakinra (10nM) (*N* = 3). – indicates no treatment. The data is displayed as Mean (± SEM) fold-expression in relation to the uninfected samples (2^−ΔΔCt^). An * indicates a significant increase in protein release compared to untreated NHBE cells (*** *p*<0.001, ** *p = *0.001–0.01), a # indicates a significant decrease in protein release/ mRNA expression of HRV14 treated samples compared to the control (*^###^ p<*0.001, *^##^ p = *0.001–0.01) and *ns* indicates not significant (*p>0.05)*.

Recombinant IL-1 is a potent activator of epithelial cells. Having shown that infection of NHBE cells with HRV triggers the release of IL-1α, IL-1β and IL-18, we investigated the extent of the contribution of these cytokines to the release of other mediators by autocrine mechanisms. NHBE cells were infected with HRV in the presence or absence of anakinra (recombinant IL-1 receptor antagonist), and Interleukin-18 binding protein (IL-18BP-Fc), which interacts with IL-18 preventing its binding to IL-18R. Addition of anakinra to the medium during and after viral infection completely blocked the release of IL-6 and IL-8 without significantly affecting IP-10 production (**Figure 4A**). In the same experiment, addition of IL-18BP-Fc had no effect on the amount of IL-6, IL-8 or IP-10 being produced by NHBE cells in response to HRV infection (**Figure 4A**) (see also Figure S1B confirming biological activity of IL-18BP-Fc). The inhibitory effect of anakinra was also observed at the transcriptional levels since an increase in IL-8 mRNA in HRV infected cells could not be detected when anakinra was present (**Figure 4B**).

**Figure 5:**
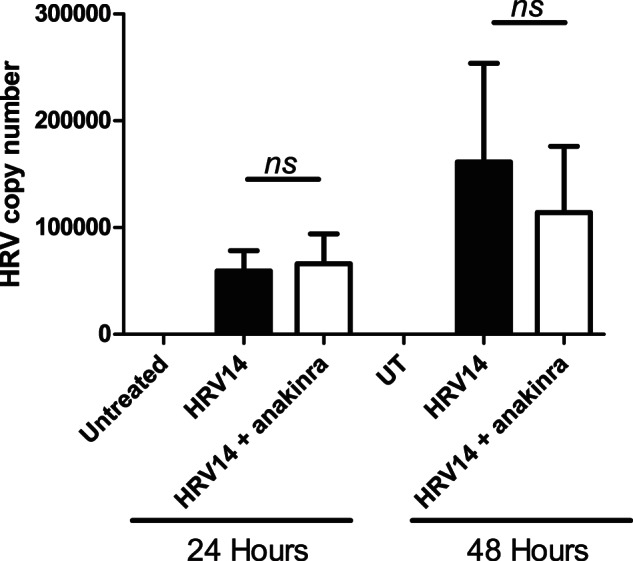
Anakinra does not inhibit viral entry or replication. NHBE cells were uninfected, infected with HRV14 or infected with HRV14 in the presence of anakinra (10nM). After the indicated time RNA was extracted and changes in viral copy number was assessed using qRT-PCR. Data is expressed as Mean ± SEM, an *ns* indicates there was no significant difference (*n*>0.05) in copy number between samples treated with HRV14 alone or HRV14 in the presence of anakinra.

Since IL-1 has been shown to positively regulate the ICAM-1 surface expression it is possible that anakinra indirectly inhibited viral entry through a reduction in ICAM-1 rather than having a direct effect on cytokine production. We assessed viral copy number by qPCR to understand this further. Virus was not detected in untreated cells. Viral mRNA was detectable 24 h after infection and appeared to be further increased at 48 h, although this was not statistically significant. Anakinra had no significant effect on these measured increases in detectable rhinovirus at both time points (**Figure 5**).

### Endogenous IL-1 signaling differently contributes to the virally-induced transcription of gene families

We then investigated the consequences of neutralising IL-1R signaling on a range of virally-induced mediators that potentially play a role in the initiation of the immune response against the virus. The effect of HRV infection on the expression of a panel of genes was determined using a qPCR / genecard approach. These genes were selected either because of their importance in IL-1 or IL-18 pathways, or they encoded pro-inflammatory mediators, type I interferons or interferon-inducible proteins, pattern recognition receptors or signaling molecules that have been previously reported to be important in viral responses by epithelial cells. Among the pro-inflammatory mediators, a significant increase in mRNA for CSF2, CSF3, CXCL2, CXCL5, IL6, IL8, and also IL1 and IL18 was observed 24 h after viral infection (**Table 1**). For most of these genes an increased transcription was still observed at the 48 h time point. Among the genes belonging to the IFN pathway, CXCL10, IFNB1, IL28A and IL29 were increased after 24 h of infection. A similar pattern was observed at 48 h except that CCL5 and CXCL11 were also significantly increased. The fold change of mRNA for IFNB1, IL28A and IL29 could not be determined as their respective mRNA could not reproducibly be detected in uninfected cells. Among the other genes analysed in this study, ICAM1 and IL1RN were the only ones significantly induced by HRV infection.

**Table 1:** IL-1 is required for the HRV-dependent release of pro-inflammatory cytokines and neutrophil chemoattractants but not T-cell chemoattractants or members of the IFN family.

		24 Hours				48 Hours			
		HRV		HRV + anakinra		HRV		HRV + anakinra	
		Fold Change vs Medium	Significance	% Inhibition vs HRV	Significance	Fold Change vs Medium	Significance	% Inhibition vs HRV	Significance
**IL-1** **Family**	*IL18*	1.5	*	28	ns	0.8	ns	−22	ns
	*IL18BP*	1.8	ns	9	ns	0.8	ns	−34	ns
	*IL18R1*	1.2	ns	2	ns	1.2	ns	−10	ns
	*IL1A*	1.9	ns	50	ns	1.6	ns	41	ns
	*IL1B*	2.4	**	57	##	1.6	ns	51	#
	*IL1R1*	1.1	ns	1	ns	1.0	ns	−2	ns
	*IL1RN*	1.7	*	48	##	1.3	ns	12	ns
**Inflammatory** **Mediators**	*CSF1*	1.3	ns	14	ns	1.4	ns	16	ns
	*CSF2*	4.2	***	89	###	4.5	***	73	###
	*CSF3*	6.0	**	92	###	4.9	*	91	###
	*CXCL2*	3.6	***	81	###	2.4	***	67	###
	*CXCL5*	7.6	**	94	###	6.8	*	92	###
	*IL15*	1.3	ns	30	ns	1.3	ns	−70	ns
	*IL6*	4.3	*	80	#	4.8	*	52	ns
	*IL8*	10.4	***	91	###	5.6	***	82	###
	*TNF*	6.0	ns	83	ns	4.6	ns	−29	ns
	*TSLP*	1.2	ns	4	ns	0.6	*	−50	ns
**Interferon** **Pathway**	*CCL5*	4.1	ns	16	ns	12.7	*	−14	ns
	*CXCL10*	21.8	*	47	ns	30.9	*	7	ns
	*CXCL11*	6.6	ns	14	ns	24.9	*	−51	ns
	*IFNAR1*	1.1	ns	14	ns	1.1	ns	−8	ns
	*IFNB1*	UR	-	0	ns	UR	-	−24	ns
	*IL28A*	UR	-	27	ns	UR	-	4	ns
	*IL29*	UR	-	40	ns	UR	-	19	ns
	*IRF3*	0.8	ns	−10	ns	1.3	ns	2	ns
	*IRF7*	3.0	ns	−29	ns	5.5	ns	−28	ns
**PPR**	*IFIH1*	2.3	ns	−10	ns	6.0	ns	−20	ns
	*LY96*	1.1	ns	11	ns	0.6	ns	2	ns
	*RARRES3*	1.0	ns	−33	ns	1.2	ns	−76	ns
	*TICAM1*	1.9	ns	−2	ns	1.5	ns	−3	ns
	*TLR3*	0.9	ns	−33	ns	1.8	ns	−83	ns
**Other**	*ICAM1*	3.0	**	74	###	2.1	*	50	ns
	*MMP3*	1.2	ns	17	ns	1.1	ns	−12	ns
	*TIMP1*	1.1	ns	−16	ns	1.1	ns	−21	ns

NHBE cells were uninfected, infected with HRV14 or infected with HRV14 in the presence of anakinra (10nM). After the indicated time RNA was extracted and changes in mRNA was determined by a microfluidic genecard. The data is expressed as Mean (± SEM) fold expression (2^−ΔΔCt^) in relation to the untreated controls. For some mediators, while HRV14 increased mRNA expression there was no detectable expression in the untreated samples (this up-regulation is indicated by UR). To assess the level of inhibition by anakinra these samples were expressed relative to the HRV14 treated samples (*N* = 3). An * indicates a significant increase in mRNA expression on treatment with HRV14 compared to untreated NHBE cells, a # indicates a significant decrease in mRNA expression when the infected NHBE cells were treated with anakinra. An *ns* indicates not significant.

*** /^# # #^ *p*<0.001, **/^# #^ *p* = 0.001–0.01, */^#^ *p = *<0.05.

**Figure 6:**
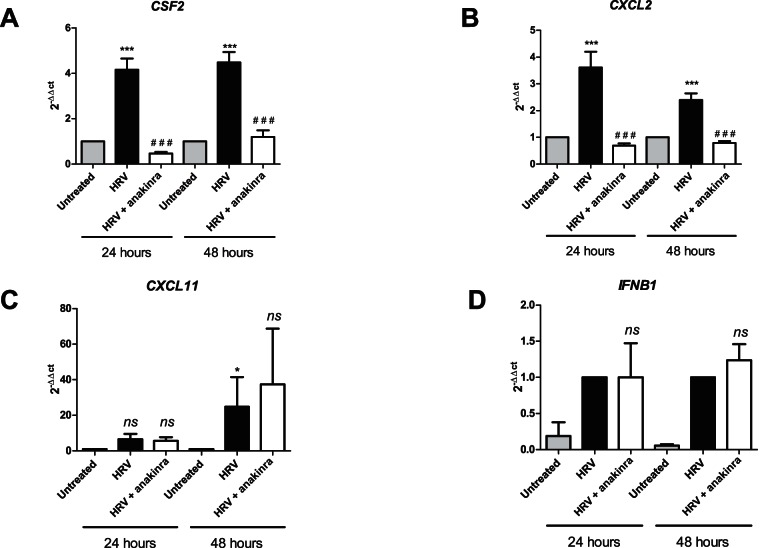
IL-1 is required for the HRV-dependent release of pro-inflammatory cytokines and neutrophil chemoattractants but not T-cell chemoattractants or members of the IFN family. NHBE cells were uninfected, infected with HRV14 or infected with HRV14 in the presence of anakinra (10 nM). After the indicated time RNA was extracted and changes in mRNA was determined by a microfluidic genecard. The data from *CSF2* (**A**), *CXCL2* (**B**), *CXCL11* (**C**) or *IFNB1* (**D**) are presented and is expressed as Mean (± SEM) fold expression (2^−ΔΔCt^) in relation to the untreated controls (**A–C**) or the HRV14 treated samples (**D**) (*N* = 3). An * indicates a significant increase in mRNA expression on treatment with HRV14 compared to untreated NHBE cells, a # indicates a significant decrease in mRNA expression when the infected NHBE cells were treated with anakinra. An *ns* indicates not significant, (***/^# # #^ *p*<0.001, **/^# #^ *p* = 0.001–0.01, */^#^ *p = *<0.05).

Whilst the gene up-regulation of the pro-inflammatory mediators highlighted above was significantly decreased at both time points by blockade of IL-1 signaling by anakinra (with the exception of IL18), there was no significant effect on the transcription of genes associated with the IFN pathway (**Table 1**). A representative data set is shown in **Figure 6** for two genes belonging to the pro-inflammatory mediator family (CSF2 and CXCL2) and 2 genes involved in the Type I interferon response (CXCL11 and IFNB1).

## Discussion

Acute exacerbations of asthma and COPD are often caused by rhinovirus infection. Structural cells such as epithelial cells are the first line of defence and play a key role in the initiation of the immune response and IL-1 family members are produced very early on during the development of the inflammatory cascade. In the present study, we have shown that IL-1α, IL-1β and IL-18 were released by NHBE cells following HRV14 infection, and that endogenous IL-1 signaling was essential for the induction and production of a number of pro-inflammatory mediators in response to the virus. Conversely, this autocrine and/or paracrine IL-1 effect on infected epithelial cells did not contribute to virally-induced IFN or IFN-driven responses.

The release of IL-1β following HRV infection of epithelial cells *in vitro* has been previously described [27], [28], [29]. IL-1β has also been measured in nasal secretions after experimental infection of healthy volunteers with rhinovirus [30]. Nevertheless, in these studies, no attempt has been made to distinguish between pro- and cleaved forms of the cytokine, nor to measure IL-1α release. Stokes *et al* [24] have demonstrated that autocrine generation of IL-1 may be important in the inflammatory response to HRV, and that knockdown of MyD88, which inhibits IL-1 signaling, can increase viral load. However, this approach does not delineate the potential contribution of each IL-1RI-activating cytokine, and MyD88 knockdown would also inhibit additional responses e.g. those mediated via TLR activation. Understanding the precise nature of the IL-1 produced, and the contributions of key IL-1 family members to the overall inflammatory response directed by the epithelial cell, allows us to deduce whether we would see the same outcome from inhibiting IL-1R1 signaling, preventing caspase 1 processing, or inhibiting either IL-1α or IL-1β separately. This knowledge is clearly helpful in determining the optimal design of a therapeutic intending to modulate virus induced inflammation via blockade of the IL-1 pathway.

Consistent with classical activation of the inflammasome by viral infection, significant levels of both IL-1β and IL-18 in their mature forms were detected in the supernatant of infected cells, and caspase-1 inhibition had an impact on this release. Mature IL-18 release by rhinovirus infected human bronchial epithelial cells has only been recently reported [28]. The amount of mature IL-18 that we detected in the supernatant of infected cells was not only higher than that reported by Shi *et al*, but also high in comparison to what is generally released by lipopolysaccharide (LPS) treated peripheral blood mononuclear cells (PBMCs) ([31] and our own unpublished data). Additionally, the data described here suggests that significant pre-stored IL-18 exists in an uncleaved pro-form in epithelial cells. These data confirm HRV as a potential trigger for significant release of mature IL-18, however inhibition of IL-18 mediated signaling had little impact on the measured autocrine responses of the epithelial cell. Although this might initially suggest little additional benefit of inhibiting caspase-1 over blockade of IL-1 signaling, other effects on epithelial cells cannot be excluded as IL-18R expression can be detected on the cells (**Figure S1A**). IL-18 would also be expected to affect other cells in the infected lung, potentially priming neutrophils [32], [33] and enhancing effector function of NK, CD4 and CD8 T cells [34]. Evidence that IL-18 may act on alternative inflammatory cells is supported by Kang *et al* (15), who showed that IL-18R−/− mice were protected from lung damage and inflammation initiated by tobacco smoke plus poly I:C or infection with influenza virus.

Although we show significant extracellular active IL-1β and IL-18, we also clearly demonstrate the presence of extracellular pro-IL-1β and pro-IL-18 after viral infection of cells. Therefore we cannot rule out that released pro-IL-1β and pro-IL-18 can also play a part in host response, via processing to active cytokines by extracellular proteases *in vivo*.

In addition to IL-1β and IL-18, we described IL-1α release following HRV infection of epithelial cells, the first report of this to our knowledge. IL-1α has been portrayed as a key signal of dysregulated cell death [21], due to its ability to be biologically active without cleavage and its additional intracellular functions. However, in this instance release of IL-1α appeared to be an active process, since inhibition of HRV-induced cytotoxicity failed to inhibit its release. Unlike the release of active IL-1β and IL-18 upon HRV infection, IL-1α release was not affected by the inhibitor YVAD, therefore its release was not dependent upon the catalytic activity of caspase-1. A recent study in myeloid cells shows that IL-1α may be actively processed intracellularly and secreted in an inflammasome-dependent or -independent fashion, depending on the nature of the stimulus [35]. Our findings that HRV infection triggers caspase-1 dependent IL-1β release and concomitant caspase-1 independent IL-1α release suggest that the findings of Groß *et al* may be more broadly applicable and merit further investigation in this context. Our data do indicate that conventional caspase-1 inhibitors, or antibodies targeting individual IL-1RI- activating cytokines may not be the optimal approach to inhibit IL-1 signaling in this situation, given that rhinovirus is capable of producing not only physiologically relevant levels of IL-1β, but also IL-1α, and there may be potential impact of extracellular cleavage of released pro-forms of IL-1β.

The signaling pathways downstream of HRV recognition have been shown to involve members of the interferon regulatory family (IRF) as well as NFkB and AP-1 (17). The resulting signaling leads to the transcription of genes belonging to the IFN pathway and of genes encoding various pro-inflammatory mediators respectively. In addition to the production of IL-1 and IL-18 we also observed the release of other pro-inflammatory mediators such as IL-6 and IL-8 as shown in previous studies [9]. At the low MOI used, although we detected an accumulation of mRNA encoding mediators belonging to the IFN pathway, there was only minimal or no release of interferon proteins into the media, although IP-10 which has been shown to be downstream of IRF signaling [36], was detectable.

We found that inhibition of IL-1RI during HRV infection using recombinant IL-1ra obliterated the production of IL-6 and IL-8, but not IP-10. These data are in accordance with recent observations in epithelial cell lines from Stokes *et al* showing that in immortalised epithelial cells (BEAS-2B) autocrine IL-1 signaling was likely to be contributing to HRV-induced cytokine production [24], and also the reported dependence of IP-10 induction on autocrine or paracrine effects of type I IFN produced by epithelial cells and monocytes during HRV infection [37]. Addressing a wider range of mediators by measuring upregulation at the gene level, we confirmed that several mediators considered to be pro-inflammatory were inhibited by blockade of IL-1RI but such inhibition was not observed for IFNs or IFN-inducible genes, confirming the pattern observed at the protein levels. MyD88 signaling has been reported to have the potential to negatively regulate IFN-signaling in HRV infection [38], but we saw no evidence IL-1RI mediated signaling was a major modifier of IFN responses in HRV-infected primary cells.

Blockade of IL-1 signaling for the duration of the HRV infection had no effect on viral titers in infected NHBE cells. Taking this together with the maintained IP-10 and IFN responses, it is unlikely inhibition of IL-1RI signaling mediates its effects by downregulation of ICAM-1 and reduced viral infection. Importantly, Stokes *et al* previously reported that knockdown of MyD88 resulted in increased viral replication in HRV-infected BEAS-2B cell line [33]. The difference in this observation may reflect either differences between primary cells and cell lines, or more likely that MyD88 knockdown will also modulate TLR signaling which may have additional effects beyond modulation of the IL-1 pathway.

In an experimental model of exacerbations in humans, when COPD patients were challenged with rhinovirus, a clear causal relationship was demonstrated between viral infection and exacerbation of disease [7]. Rhinovirus infection was temporally associated with a significant and sustained neutrophil influx and increased levels of neutrophil elastase and IL-8 in the sputum of subjects with COPD. There were strong correlations between sputum neutrophil elastase levels and severity of symptoms and airflow obstruction exclusively in subjects with COPD, not in subjects matched for smoking history but without COPD. In addition, virus load correlated significantly with neutrophil numbers and inflammatory mediators in serum, sputum, and BAL. Therefore, therapies that reduce or normalise neutrophil response to viral infection without reducing anti-viral responses could be beneficial in reducing the severity of AECOPD, although appropriate consideration of potential effects on bacterial host defence in colonised patients would be required. Given the neutrophil influx is rapid in response to infection, and in patients a viral infection only becomes evident on onset of symptoms, any such therapy likely would require prophylactic administration for maximum efficacy, although some benefit of treatment at onset of symptoms may be evident if the sustained neutrophil influx is important in severity and/or duration of symptoms. Although we have studied the impact of IL-1RI blockade in HRV responses of epithelial cells without assessing the impact on other structural or tissue-associated cells in the lung, our data could suggest that in the context of a virally-triggered exacerbation of COPD, blocking IL-1 signaling may translate into a decreased inflammatory response, e.g. lung neutrophilia, with limited effect on the induction of anti-viral mediators downstream of the IFN pathway and the recruitment of effectors involved in the adaptive immune response such as CD8 T cells. This has not been addressed *in vivo* with rhinovirus, however IL-1R1−/− mice infected with influenza virus demonstrated a decreased lung neutrophilia and pulmonary inflammation compared to wild type mice [39]. In this model, IL-1 signaling was found to be important for the priming of virus-specific CD4 T cells but, at the peak of the lymphocyte response, the number of influenza virus specific cytotoxic T cell responses was comparable in wild type and IL-1R1−/− and the virus titer only moderately increased in the latter. Smoke exposure can also exacerbate the response to influenza A virus in mice, and IL-1R deficiency has been shown to attenuate exacerbated neutrophilic responses in smoke-exposed influenza-infected animals [40]. Interestingly, this effect could be recapitulated with an anti-IL-1α antibody, while IL-1RI−/− and anti-IL-1α-treated mice did not suffer from an increase in viral burden.

Updated new guidelines from regulatory agencies suggest modification or prevention of exacerbations is an acceptable key aim of new medicinal products for the treatment of COPD. Given the importance of common viruses in induction of exacerbations in chronic respiratory disease further study of inhibition of the IL-1 pathway should be undertaken. Given the data presented here showing their combined involvement in HRV infection, agents that block both IL-1α and IL-1β, such as IL-1RI antagonists, may be appropriate as an anti-inflammatory in the treatment of COPD exacerbations. A phase II clinical study with an IL1R antagonist (NCT01448850) is currently underway in patients with COPD that should further clarify the potential role of this pathway in human respiratory disease.

## Supporting Information

Materials and Methods S1 (DOC)

Figure S1 **IL-18 receptor expression and inhibitory activity of IL-18BP.** (**A**) NHBE cells from 2 separate donors were labelled with the indicated anti IL-18R antibody or isotype control. The data shows the mean fluorescent intensity (MFI). Representative staining is shown from 2 independent experiments. (**B**) KG-1 cells were treated with or without 10ng/mL rIL-18 in the presence of the indicated inhibitor (− indicates no treatment, IL-18BP and the control IgG were both used at 33nM). Following a 24 hour incubation the release of IFNγ into the medium was measured by ELISA. The data shows mean +/− SD (*n* = 1).
(TIF)

Figure S2 **Mediator release at different MOI of virus.** NHBE cells were infected with HRV14 at the indicated MOI. After the indicated time the levels of IL-1β (A) and IL-8 (B) released into the media was measured by ELISA. The data is from a representative experiment and shows mean +/− SD mediator release.
(TIF)

Table S1 **MOI calculation.**
(DOC)
