# Supplementary material for: The Role of Interleukin-1 and Interleukin-18 in Pro-Inflammatory and Anti-Viral Responses to Rhinovirus in Primary Bronchial Epithelial Cells
Source: PLoS One. 2013 May 28;8(5):e63365. doi: 10.1371/journal.pone.0063365 (PMC3665753; doi:10.1371/journal.pone.0063365)
Supplement: Materials and Methods S1 — (DOC) [file pone.0063365.s001.doc]

**SUPPLEMENTARY MATERIALS AND METHODS.**

**IL-18R expression**.

NHBE cells were stained with a fixable viability dye (efluor 780; eBioscience, Hatfield, UK) before being incubated with the following antibodies; mouse anti human IL-18Rα-PE (R&D systems, Minneapolis, MN), Mouse IgG1-PE isotype control (BD bioscience, Oxford, UK), mouse anti human IL-18Rβ (R&D systems), mouse IgG2b isotype control (eBioscience) or Goat anti mouse-PE (Jackson ImmunoResearch Laboratories, Newmarket, UK). Following staining the cells were washed, the level of bound antibody measured on a BD FACSCanto II and the data were analysed using FlowJo. Dead cells (with a high viability dye signal) were excluded from the analysis.

**Inhibition of IL-18 signalling with IL-18BP.**

KG-1 cells (European Collection of Cell Culture (ECACC, Health Protection Agency, Porton Down, UK) were seeded at 1x10^5^ cells/well in a 96 well plate in complete media (IMDM supplemented with 5% foetal calf serum and penicillin/streptomycin [Invitrogen, Paisley, UK]) containing the indicated rIL-18BPa-Fc Chimera (R&D systems) or isotype control. Following stimulation with rIL-18 (R&D systems) the cells were incubated for 24 hours before the media was collected and the levels of IFNγ determined by ELISA (BD Bioscience).

**Derivation of the Multiplicity of Infection (MOI)**

To determine the concentration of infectious particles in a batch of virus HELA-Ohio cells were infected with the indicated dilution of HRV14. Following incubation the cells were stained with crystal violet and the OD was measured (Figure S2Ai). Any reading that was < 80% of the mean OD of non-infected cells was scored as having cytopathic effects (CPE) (1 indicates a well scored positive for cytopathic effects) (Figure S2 Aii). The TCID50 was determined using the Spearman Kärber method;

TCID50= Highest dilution giving 100% CPE +½ - total number of test units showing CPE

number of test units per dilution

For the example shown in Figure SA2ii.

-5 + 1/2 - 8/6 = -5.83

= 105.8TCID­­50­­ units/0.1 mL

=106.8TCID­­50­­ units/mL.

=6.8x10^6^ TCID50 units/mL

6.8x10^6^ * 0.7 = 4.8 x10^6^ theoretical Plaque Forming Units (PFU) /mL

A mean (+/- SEM) theoretical PFU of 7.9x10^6^/mL (+/- 2.1x10^6^) was derived from 7 independent experiments.

The Theoretical Plaque Forming Units was then used to calculate the MOI; the number of infective particles added to the NHBE cells.

MOI= theoretical PFU/ dilution factor/ number of cells* volume added.

7.9 x10^6^ /4000/1.2x105*0.4= **0.007**
