# Supplementary figures and images for: The Role of Interleukin-1 and Interleukin-18 in Pro-Inflammatory and Anti-Viral Responses to Rhinovirus in Primary Bronchial Epithelial Cells
Source: PLoS One. 2013 May 28;8(5):e63365. doi: 10.1371/journal.pone.0063365 (PMC3665753; doi:10.1371/journal.pone.0063365)

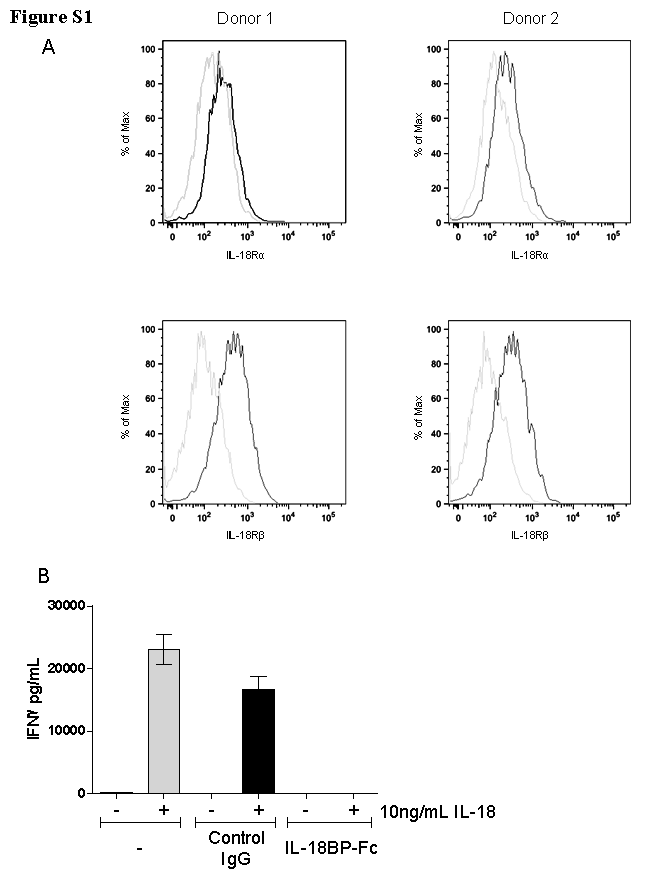

Supplement: Figure S1 — IL-18 receptor expression and inhibitory activity of IL-18BP. (A) NHBE cells from 2 separate donors were labelled with the indicated anti IL-18R antibody or isotype control. The data shows the mean fluorescent intensity (MFI). Representative staining is shown from 2 independent experiments. (B) KG-1 cells were treated with or without 10ng/mL rIL-18 in the presence of the indicated inhibitor (− indicates no treatment, IL-18BP and the control IgG were both used at 33nM). Following a 24 hour incubation the release of IFNγ into the medium was measured by ELISA. The data shows mean +/− SD (n = 1). (TIF) [file pone.0063365.s002.tif]
