# Supplementary material for: The Role of Interleukin-1 and Interleukin-18 in Pro-Inflammatory and Anti-Viral Responses to Rhinovirus in Primary Bronchial Epithelial Cells
Source: PLoS One. 2013 May 28;8(5):e63365. doi: 10.1371/journal.pone.0063365 (PMC3665753; doi:10.1371/journal.pone.0063365)
Supplement: Table S1 — MOI calculation. (DOC) [file pone.0063365.s004.doc]

***Table S1***

|  | Virus dilution |  |  |  |  |  |  |  |  |  |
| --- | --- | --- | --- | --- | --- | --- | --- | --- | --- | --- |
|  |  |  |  |  |  |  |  |  |  |  |
|  | -3 | -4 | -5 | -6 | -7 | -8 | -9 | -10 | -11 | 0 |
| Replicate | 0.10 | 0.11 | 0.42 | 0.64 | 0.89 | 0.87 | 0.84 | 0.86 | 0.86 | 0.84 |
|  | 0.10 | 0.11 | 0.36 | 0.33 | 0.87 | 0.87 | 0.83 | 0.85 | 0.86 | 0.84 |
|  | 0.10 | 0.11 | 0.41 | 0.72 | 0.89 | 0.93 | 0.87 | 0.85 | 0.86 | 0.84 |
|  | 0.11 | 0.11 | 0.51 | 0.87 | 0.86 | 0.91 | 0.87 | 0.87 | 0.85 | 0.84 |
|  | 0.10 | 0.11 | 0.62 | 0.86 | 0.88 | 0.87 | 0.85 | 0.85 | 0.86 | 0.83 |
|  | 0.10 | 0.11 | 0.46 | 0.84 | 0.89 | 0.86 | 0.85 | 0.88 | 0.91 | 0.85 |
|  |  |  |  |  |  |  |  |  |  |  |
|  | -3 | -4 | -5 | -6 | -7 | -8 | -9 | -10 | -11 | 0 |
| Replicate | 1 | 1 | 1 | 1 | 0 | 0 | 0 | 0 | 0 | 0 |
|  | 1 | 1 | 1 | 1 | 0 | 0 | 0 | 0 | 0 | 0 |
|  | 1 | 1 | 1 | 0 | 0 | 0 | 0 | 0 | 0 | 0 |
|  | 1 | 1 | 1 | 0 | 0 | 0 | 0 | 0 | 0 | 0 |
|  | 1 | 1 | 1 | 0 | 0 | 0 | 0 | 0 | 0 | 0 |
|  | 1 | 1 | 1 | 0 | 0 | 0 | 0 | 0 | 0 | 0 |

***MOI calculation.*** Representative data (from 7 independent experiments) is shown for HeLa Ohio Cells that were infected as outlined in the materials and methods. The OD of each replicate was measured following staining with crystal violet **(A)**. The OD was used to score for cytopathic effects (CPE). 1 indicates CPE was present in a single replicate while 0 indicates no CPE were observed **(B)**.
